# Poly(3-hydroxybutyrate) Modified by Plasma and TEMPO-Oxidized Celluloses

**DOI:** 10.3390/polym12071510

**Published:** 2020-07-07

**Authors:** Denis Mihaela Panaitescu, Sorin Vizireanu, Sergiu Alexandru Stoian, Cristian-Andi Nicolae, Augusta Raluca Gabor, Celina Maria Damian, Roxana Trusca, Lavinia Gabriela Carpen, Gheorghe Dinescu

**Affiliations:** 1 Polymer Department, National Institute for Research and Development in Chemistry and Petrochemistry, 202 Spl. Independentei, 060021 Bucharest, Romania; stoian.sergiu@gmail.com (S.A.S.); ca_nicolae@yahoo.com (C.-A.N.); ralucagabor@yahoo.com (A.R.G.); 2 National Institute for Laser, Plasma and Radiation Physics, Atomistilor 409, Magurele-Bucharest, 077125 Ilfov, Romania; lavinia.carpen@infim.ro (L.G.C.); dinescug@infim.ro (G.D.); 3 Advanced Polymers Materials Group, University Politehnica of Bucharest, 1-7 Polizu Street, 011061 Bucharest, Romania; c.damian@tsocm.pub.ro; 4 Science and Engineering of Oxide Materials and Nanomaterials, University Politehnica of Bucharest, 1-7 Gh. Polizu Street, 011061 Bucharest, Romania; truscaroxana@yahoo.com; 5 Faculty of Physics, Bucharest University, 405 Atomistilor Street, Magurele-Bucharest, 077125 Ilfov, Romania

**Keywords:** cellulose, plasma in liquids, polyhydroxyalkanoate, polymer composites, thermal properties, DMA

## Abstract

Microcrystalline cellulose (MCC) was surface modified by two approaches, namely a plasma treatment in liquid using a Y-shaped tube for oxygen flow (MCC-P) and a TEMPO mediated oxidation (MCC-T). Both treatments led to the surface functionalization of cellulose as illustrated by FTIR and XPS results. However, TEMPO oxidation had a much stronger oxidizing effect, leading to a decrease of the thermal stability of MCC by 80 °C. Plasma and TEMPO modified celluloses were incorporated in a poly(3-hydroxybutyrate) (PHB) matrix and they influenced the morphology, thermal, and mechanical properties of the composites (PHB-MCC-P and PHB-MCC-T) differently. However, both treatments were efficient in improving the fiber–polymer interface and the mechanical properties, with an increase of the storage modulus of composites by 184% for PHB-MCC-P and 167% for PHB-MCC-T at room temperature. The highest increase of the mechanical properties was observed in the composite containing plasma modified cellulose although TEMPO oxidation induced a much stronger surface modification of cellulose. This was due to the adverse effect of more advanced degradation in this last case. The results showed that Y-shaped plasma jet oxidation of cellulose water suspensions is a simple and cheap treatment and a promising method of cellulose functionalization for PHB and other biopolymer reinforcements.

## 1. Introduction

Biopolymers are seen as a viable alternative to synthetic polymers in many applications and they are unparalleled in the medical field [1,2,3]. Poly(3-hydroxybutyrate) (PHB) is an aliphatic polyester obtained by microbial synthesis and, for the moment, is the most affordable of polyhydroxyalkanoates (PHAs) family. The known shortcomings of PHB are the high cost and brittleness along with the low thermal stability during melt processing [4,5]. Adding plasticizers, elastomers, and organic or inorganic fillers are the most used strategies to improve PHB properties [6,7,8]. Among the fillers, micro- and nano-celluloses were most intensely studied to modify PHB and other aliphatic polyesters [9,10,11,12].

Microfibrillated cellulose is obtained from cellulose, a sustainable and low-cost naturally occurring biopolymer. This is the most abundant organic polymer on Earth and comprises 33% of all the plants on the planet [13,14]. Microfibrillated cellulose is biodegradable and biocompatible and has reduced carbon dioxide emissions in the environment. It shows an adaptable surface chemistry and a unique spectrum of properties: high water-uptake capability, low density, high crystallinity and Young’s modulus, and good thermal stability [12,13,14]. The research, production, and application of microfibrillated cellulose have experienced a real boost in the last twenty years. It can be extracted from a multitude of sources, such as wood, plants, algae, by-products, and waste of the agro-food industry by mechanical disintegration [13,14,15]. Refining, homogenization, high power ultrasonication, or microfluidization, with or without additional enzymatic or chemical pretreatments are generally the most used methods to obtain long flexible microfibrils [12,13,14,15].

TEMPO-mediated oxidation of cellulose consists of surface oxidation of cellulose in aqueous suspension using sodium hypochlorite as oxidant and 2,2,6,6-tetramethylpiperidine-1-oxyl radical (TEMPO) and sodium bromide as catalysts at an alkaline pH [16,17,18]. This pre-treatment proved to be a successful method for ensuring the selective conversion of C6 hydroxyl group of cellulose to carboxylate group. Previous studies have shown the great advantage of this pre-treatment which reduces the energy consumption and the number of cycles in the subsequent stages of mechanical defibrillation [18,19].

Recently, plasma treatment was found to be efficient for the defibrillation and functionalization of cellulose [20,21]. Plasma treatment was mainly studied to improve the surface of cellulose textiles or membranes but, in certain conditions, plasma treatment may ensure the defibrillation and chemical functionalization of cellulose [21]. In particular, a plasma torch immersed in the water suspension of cellulose in the presence of different reactive gases induced physical and chemical changes of cellulose [21] and is proposed as an alternative to the mechano-chemical methods usually employed for the modification of cellulose and fabrication of nanocellulose. Moreover, plasma treated cellulose showed a good reinforcing effect in PHB/cellulose composites, increasing both the Young’s modulus and tensile strength [21].

Plasma treatment of nanocellulose water suspensions using a filamentary plasma jet based on dielectric barrier discharge (DBD) proved to be very advantageous in the surface functionalization of nanocellulose [20]. Only 0.2 wt % of filamentary jet plasma functionalized nanocellulose increased the tensile strength and Young’s modulus of PHB nanocomposite by 10–15% compared to the nanocomposite with the same amount of untreated nanocellulose. The surface functionalization of cellulose in water suspensions using DBD plasma is an environmentally friendly, simple, cheap, and low-energy consuming treatment that overcomes the disadvantages of complex, time-consuming, and energy-intensive chemical-mechanical treatments. Moreover, toxic products that must be deactivated are generally released from chemical treatments. However, DBD filamentary plasma sources cannot work with higher fluxes of reactive gases. Our preliminary attempts have shown that a flux of reactive gas (O_2_ or NH_3_) greater than 20 sccm (3.33 × 10^−7^ standard cubic meter per second) led to the extinction of the discharge submersed in the water suspensions of cellulose. For this reason, in this work, the DBD plasma source was modified for the addition of the reactive gas laterally in the discharge. This new method makes it possible to work with much higher fluxes of oxygen or other reactive gases, even greater than 500 sccm.

In this study, a microcrystalline cellulose (MCC) was treated with a filamentary Y-shaped DBD plasma jet generated in argon-oxygen gas mixture and immersed in the liquid suspension of cellulose. For a comparison with chemical treatments, MCC was chemically modified using TEMPO mediated oxidation, an eco-friendlier chemical method compared to acid hydrolysis. The changes induced by the two treatments (plasma and TEMPO) were highlighted by Fourier transform infrared (FTIR) spectroscopy, X-ray photoelectron spectroscopy (XPS), and thermogravimetric analysis (TGA). Plasma and TEMPO modified celluloses (MCC-P and MCC-T) were used to reinforce a PHB matrix and the composites were characterized by thermal analyses, dynamic mechanical analysis, and scanning electron microscopy (SEM).

## 2. Materials and Methods

### 2.1. Materials

Microcrystalline cellulose (MCC) was purchased from Sigma-Aldrich (Saint Louis, MO, USA). MCC is rod-shaped and has a mean diameter of 20 µm, a bulk density of 0.5 g/cm^3^ and a small aspect ratio. PHB type P304 from Biomer (Schwalbach am Taunus, Germany) was used to prepare the composites. A tensile strength of 28 MPa, determined according to ISO 527:2012 standard with 50 mm/min [22], was provided for PHB type P304 by the producer. Acetonitrile 99% (ACN) purchased from Fluka Chemie AG (Buchs, Switzerland) was used as received. Further, 2,2,6,6-tetramethylpiperidine-1-oxyl radical, 98% (TEMPO) was purchased from Sigma-Aldrich (Saint Louis, MO, USA), sodium hypochlorite 10% from Oltchim (Rm-Valcea, Romania), sodium bromide, hydrochloric acid, and sodium hydroxide, all of analytical grade, were obtained from Merck KGaA (Darmstadt, Germany).

### 2.2. Plasma Treatment of Cellulose Suspensions

**Figure 1:**
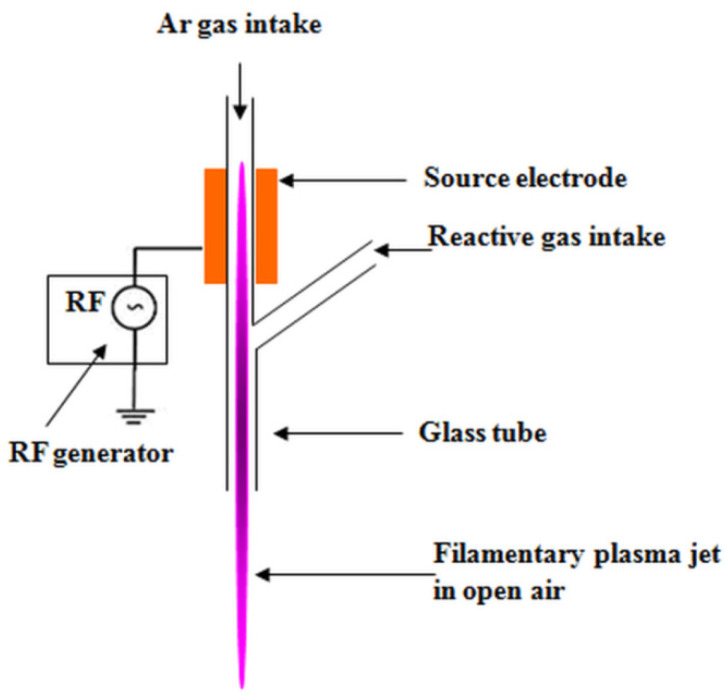
Configuration of the DBD Y-shaped source.

The cellulose suspension (5 wt %) was prepared by dispersing 5 g MCC in 95 mL of distilled water by ultrasonication using a Elmasonic S 15 H bath (Elma, Singen, Germany) for 1 h. The treatment of MCC suspensions was carried out using a DBD plasma source with floating electrode, coupled with a radiofrequency power supply [23,24]. The discharge tube was modified in a Y-shape geometry (Figure 1). The Y-shaped configuration allows for the introduction of large amounts of reactive gases without extinguishing the discharge.

The plasma jet was initiated in argon (RF power 100 W, flow rate 3000 sccm), in open ambient conditions, and then immersed in the water suspension of MCC. Sccm, standard cubic centimeters per minute, is a measure for the flow rate of particles, 1 sccm denotes that in one minute in the chamber entered a number of particles corresponding to those existing in a volume of 1 cubic centimeter at standard temperature (273 K) and pressure (1 atm). An oxygen flux of 500 sccm was introduced through the lateral branch of the Y-tube and the discharge was maintained for 1 h of treatment. Then, ACN (30%) was mixed in the water suspension of MCC for ensuring better conditions for surface functionalization of cellulose and the suspension was plasma treated with Ar for 30 min using the same source. Plasma treated MCC suspension was ultrasonicated for 30 min and then freeze dried for 72 h using a FreeZone 2.5 L Benchtop Freeze Dry System (Labconco, Kansas City, MO, USA).

### 2.3. TEMPO Mediated Oxidation of MCC

MCC was oxidized using 2,2,6,6-tetramethylpiperidine-1-oxyl radical as a catalyst and sodium hypochlorite as an oxidant. Five grams of MCC were dispersed in 195 mL of distilled water by magnetic stirring at room temperature for 24 h. TEMPO (0.08 g corresponding to 0.1 mmol/g cellulose) was dispersed in 100 mL of distilled water until complete dissolution using an Elmasonic S40 ultrasonic bath (Elma, Germany). Meanwhile, 0.52 g NaBr (1.0 mmol/g cellulose) were added over the TEMPO solution and dispersed using the same bath. The TEMPO/NaBr solution was added to the cellulose suspension in small portions under magnetic stirring. Then, 10% NaClO solution (29.80 g corresponding to 8.0 mmol/g cellulose) was added dropwise for half an hour and stirred for 3h from the moment of the first drop of NaClO. The pH of the solution was kept constant (pH 10–11) during the reaction by the addition of 1M solutions of HCl and NaOH. After the completion of the reaction, the TEMPO treated cellulose suspension was dialyzed using Spectra/Por molecular porous membrane (MWCO 3.5 kDa, SpectrumLabs (Rancho Dominguez, CA USA) for removing the unreacted products. After the treatments, the MCC suspension was ultrasonicated for 30 min and then freeze dried for 72 h using a FreeZone 2.5 L Benchtop Freeze Dry System (Labconco, Kansas City, MO, USA). Plasma treated and TEMPO oxidized MCC were denoted as MCC-P and MCC-T.

### 2.4. Preparation of PHB Composites with Untreated, Plasma and TEMPO Oxidized MCC

**Figure 2:**
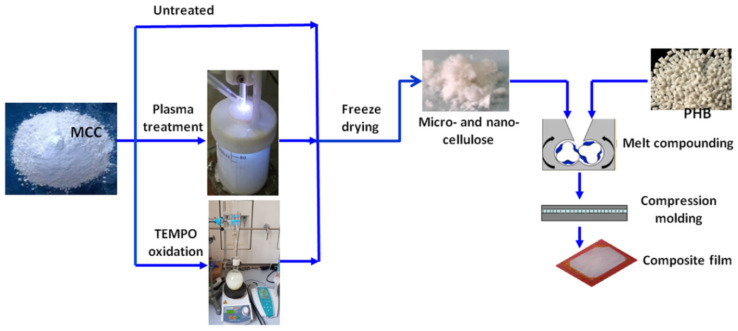
The sequence of main operations for the fabrication of PHB composites with untreated, plasma treated and TEMPO oxidized MCC.

PHB pellets, MCC-P and MCC-T were dried in vacuum ovens at 60 °C for 4 h. Different treated MCC (2 wt %) was melt blended with PHB using a Brabender LabStation (Duisburg, Germany) with a mixing chamber of 30 cm^3^, at 165 °C for 8 min at a rotor speed of 50 min^−1^. For morphological and mechanical characterization, composites sheets of 0.5 mm in thickness were obtained by compression molding using an electrically heated press (P200E, Dr. Collin, Maitenbeth, Germany). The composites were pressed at 175 °C for 120 s preheating (without pressure), 75 s under pressure (100 bar), and cooling for 1 min in a cooling cassette. The sheets were kept at room temperature for at least 2 weeks before characterization. PHB composites with untreated MCC, MCC-T, and MCC-P were denoted as PHB-MCC, PHB-MCC-T, and PHB-MCC-P. The schematic representation of operations for obtaining PHB composites with different treated MCC is shown in Figure 2.

### 2.5. Characterization

#### 2.5.1. SEM Investigation

The compression molded sheets of PHB–MCC composites were fractured in liquid nitrogen and then sputter-coated with a thin layer of gold. The surface of fractured composites was analyzed by SEM using a Quanta Inspect F scanning electron microscope (FEI-Philips, Hillsboro, OR, USA) equipped with a field emission gun. The microscope was operated in high vacuum at 30 kV with a resolution of 1.2 nm.

#### 2.5.2. Chemical Characterization by FTIR and XPS

The FTIR spectra of untreated, plasma, and TEMPO treated cellulose were recorded on a Tensor 37 spectrometer from Bruker Optics (Ettlingen, Germany) with an attenuated total reflectance (ATR) accessory. The spectra were collected in duplicate, at room temperature, from 400 to 4000 cm^−1^, with 16 scans at a resolution of 4 cm^−1^. Plasma treated and TEMPO oxidized cellulose suspensions were dropwise deposited on silicon wafers and dried several days at room temperature to obtain films. The surface of cellulose films was characterized by X-ray photoelectron spectroscopy (XPS) using ESCALAB™ XI+ spectrometer (Thermo Scientific, Waltham, MA, USA) with a monochromatic Al Kα source at 1486.6 eV. Both survey spectra (step of 1 eV) at a pass energy of 100 eV and high-resolution spectra in the C 1s and O 1s regions (step of 0.1 eV) at pass energy of 20 eV were recorded.

#### 2.5.3. Thermal Characterization

Thermogravimetric analysis (TGA) was used to characterize the thermal stability of MCC samples before and after the treatments and the thermal behavior of PHB composites with different treated celluloses. TGA was carried out on duplicate cellulose samples sealed in aluminium pans using a SDT Q600 V20.9 (TA Instruments Inc., New Castle, DE, USA) with helium as the purge gas (100 mL/min). TGA measurements of PHB composites were carried out on a TGA Q500 V20.10 (TA Instruments Inc., New Castle, DE, USA) in platinum pans, using nitrogen as purge gas at a flow rate of 90 mL/min. All the samples, celluloses or composites, were heated from 25 to 700 °C at a heating rate of 10 °C/min.

#### 2.5.4. Differential Scanning Calorimetry (DSC)

DSC measurements were carried out on composites using a DSC Q2000 V24.9 from TA Instruments (New Castle, DE, USA) under helium flow (30 mL/min). Samples of 6–7 mg were heated from 30 to 200 °C and equilibrated for 3 min for erasing the thermal history (first heating cycle), cooled down to −50 °C, isothermal for 2 min (cooling cycle) and reheated to 200 °C (second heating cycle). A heating/cooling rate of 10 °C/min was used in all cycles. The degree of crystallinity (*X_c_*) was determined from the second heating cycle by dividing the total melting enthalpy Δ*H_m_* (of the peak and shoulder) by the amount of PHB in the composites (*W_PHB_*) and Δ*H*_0_, the melting enthalpy of 100% crystalline PHB (146 J/g [25]):

(1) $$X_{c}(\%)=\frac{\Delta H_{m}}{\Delta H_{0}}\cdot \frac{100}{W_{PHB}}$$

#### 2.5.5. DMA Characterization

The dynamic mechanical properties of composites were determined using a DMA Q800 (TA Instruments, New Castle, DE, USA) in multi-frequency-strain mode. Bar specimens of 12 × 6 × 0.5 mm^3^ were cut from the composite sheets and cooled to −35 °C, kept isothermally at this temperature for 5 min, and heated to 165 °C with a heating rate of 3 °C/min. Storage modulus, showing the recoverable stored energy and tan δ or damping factor, which is the ratio of the loss modulus to the storage modulus, were plotted against temperature.

## 3. Results and Discussion

### 3.1. Characterization of MCC, MCC-P and MCC-T

The changes induced in cellulose by the two treatments (plasma and TEMPO) were highlighted by TGA, FTIR, and XPS.

#### 3.1.1. Effect of the Treatments on the Thermal Stability of MCC

**Figure 3:**
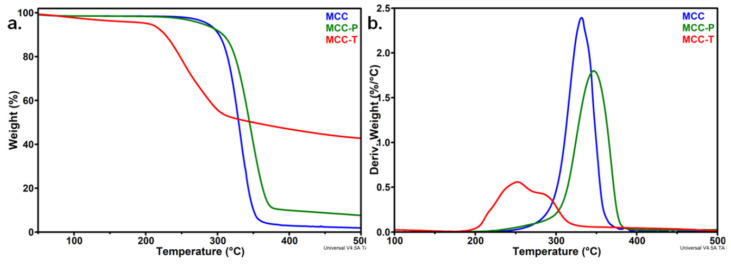
TGA (**a**) and DTG curves (**b**) for microcrystalline cellulose (MCC), TEMPO oxidized cellulose (MCC-T) and plasma treated cellulose (MCC-P).

TGA and derivative thermogravimetric (DTG) curves of MCC, MCC-T, and MCC-P are shown in Figure 3. Pure MCC showed one major degradation step with the onset degradation temperature (*T_on_*) at 309.3 °C and the temperature of the maximum degradation rate (*T_d_*) at 331.5 °C, similar to other observations [21,26]. The highest loss of weight was noticed between 280 and 360 °C, when the reactions of decomposition, depolymerization, and decarboxylation overlap [26].

The thermal stability of MCC was significantly influenced by TEMPO-mediated oxidation and only small changes were observed after the plasma treatment (Figure 3). The degradation started at a much lower temperature of 222 °C in the case of MCC-T instead of 309 °C for pristine MCC. Similarly, the *T_d_* decreased by 80 °C, from 331.5 to 251.9 °C. These changes are due to the oxidation reactions induced by the TEMPO treatment [27,28]. A similar decrease of the onset degradation temperature, with about 100 °C, was reported by Fukuzumi et al. in the case of TEMPO oxidized bleached kraft pulp fibers and explained by the formation of sodium carboxylate groups at the C6 primary hydroxyls on the surface of cellulose crystals and in the disordered regions [27]. Saito and Isogai reported an important decrease of the degree of polymerization (DP) in the case of TEMPO-modified cellulose cotton linter [16]. The decrease of DP, related to a lower average molecular weight and a lower thermal stability, was explained by the oxidation of all hydroxyl groups of cellulose and the formation of C6 carboxylate or aldehyde groups and C2 or C3 ketones [29]. The high broadness of the DTG peak in the case of MCC-T shows the overlap of at least two degradation processes. The first peak, at about 222 °C, was determined by the degradation of more unstable anhydroglucuronate units resulting from TEMPO-oxidation, and the second at a temperature of 285 °C, lower than the *T_d_* of unmodified cellulose, may be ascribed to the decomposition of unmodified or slightly oxidized cellulose influenced by the adjacent sodium carboxylate groups [28].

A completely different behavior was observed after the plasma treatment of MCC (Figure 3): *T_on_* increased by 9 °C and *T_d_* by 15 °C compared to untreated MCC. A similar increase of the maximum degradation temperature was reported for nanocellulose treated with a DBD plasma source in Ar or Ar/N_2_ flow [20]. The better thermal stability of MCC-P compared to untreated MCC resulted from the removal of low molecular weight fractions or impurities from the MCC suspended in water under the influence of submerged plasma discharge [20]. The different charred residue values at 500 °C (R_500_), 7.6% for MCC-P, 42.7% for MCC-T, and only 1.9% for MCC, also suggest different degradation processes after the treatments. Cellulose decomposed almost completely in inert atmosphere and the residue is very low for untreated MCC [29]. The large charred residue value of MCC-T, 22 times higher than that of MCC, may be related to the decomposition products of sodium anhydroglucuronate units. Furthermore, carboxylate, aldehyde, or ketone groups of MCC-T may be involved in a great number of condensation, grafting, or crosslinking reactions, resulting in an important amount of highly condensed polycyclic aromatic structures [21,30]. Moreover, the decreased DP supposed in the case of MCC-T may lead to an increased number of chain ends, which decompose at a lower temperature giving an increased amount of char [31]. Therefore, the smaller R_500_ value of MCC-P compared to MCC-T, only four times higher than that of MCC, may be related to a much lower amount of oxidized cellulose after the plasma treatment. FTIR analysis may give more information on the chemical modifications induced by the treatments on the surface of cellulose.

#### 3.1.2. Surface Chemical Properties of Modified Celluloses

**Figure 4:**
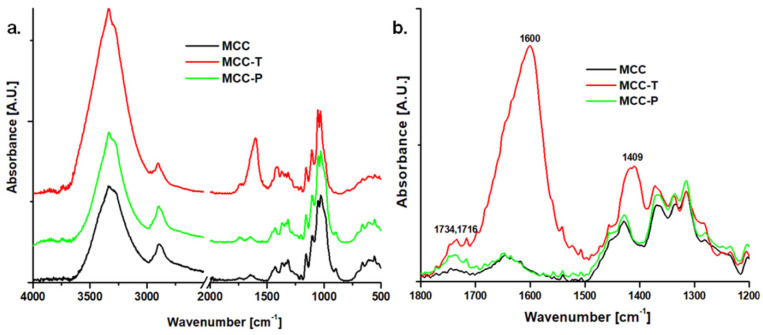
FTIR spectra of MCC, MCC-T and MCC-P (**a**); FTIR spectra in the range from1800 to 1200 cm^−1^ (**b**).

The FTIR spectra of different treated MCC are shown in Figure 4a. FTIR spectrum of MCC shows the characteristic vibrations of cellulose: (i) 3000–3600 cm^−1^ due to the O–H stretching vibrations of hydrogen bonded hydroxyl groups, (ii) 2800–3000 cm^−1^ due to asymmetric and symmetric stretching vibrations of C–H, (iii) at 1645 cm^−1^ due to the O–H bending vibration in bound water, (iv) multiple bands between 1275 and 1435 cm^−1^ due to the bending and deformation vibrations of C–H and CH_2_, (v) at 1162 and 897 cm^−1^ due to the C–O–C stretching vibrations at the β-glucosidic linkage, and (vi) at 1054 and 1030 cm^−1^ due to the C–O stretching vibrations [26,32,33].

Major changes were noticed in the FTIR spectrum of MCC-T (Figure 4). A new and intense peak appeared at 1600 cm^−1^ which is characteristic of asymmetric C=O stretching vibrations in COO¯ of sodium carboxylate [34,35]. A new and broad band appeared at 1409 cm^−1^ due to the symmetric stretching vibrations of COO⁻ [34]. In addition, two new bands were noticed in MCC-T at 1716 and 1734 cm^−1^ (Figure 4b), which may be associated to the C=O stretching vibrations in carboxylic acid [27,36,37].

Small changes were observed between the FTIR spectra of MCC-P and untreated MCC (Figure 4a,b). A broad band at 1737 cm^−1^ and a second band at 1716 cm^−1^ were noticed in MCC-P similar to MCC-T, and they are assigned to the C=O stretching vibrations in carboxylic acid. The appearance of the new bands around 1734/1737 cm^−1^ indicates the oxidation of MCC by both TEMPO and plasma treatments [36]. The degree of oxidation (DO) may be estimated from the FTIR spectra by comparing the intensity of the band near 1735 cm^−1^ with that of the band at 1050 cm^−1^ which comes from the cellulose backbone [36]. Different DO values were obtained for MCC-T and MCC-P, 0.06% and 0.12%. They correlate with the different oxidation intensity induced by the treatments. Similar DO values were reported for TEMPO-mediated oxidation of cellulose whiskers and cellulose from pulp residue in comparable reaction conditions [34,36].

**Figure 5:**
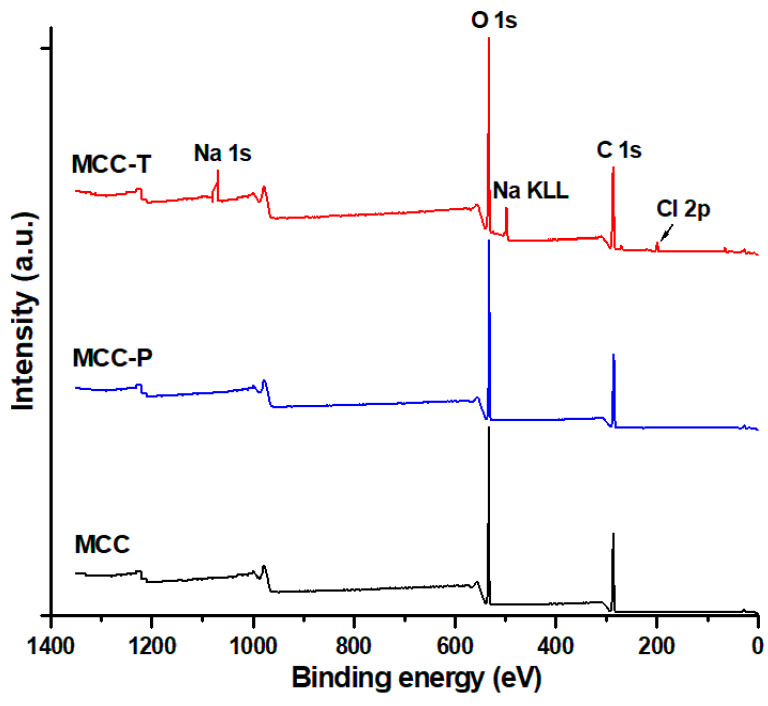
XPS survey spectra of different treated MCC.

For a better understanding of the chemical changes on the surface of MCC after the treatments, untreated and treated cellulose was also characterized by XPS. Survey spectra are shown in Figure 5. XPS survey scans indicate the presence of carbon at 286.3 eV and oxygen at 533.3 eV in all samples and, in addition, chlorine (199.3 eV) and sodium (1072.3 eV) [38] in MCC-T. The O KLL Auger peak was also noticed in all survey spectra at 978 eV. The O/C ratio differed slightly before and after treatments (Table 1) and it was close to the theoretical ratio for pure cellulose (0.83). It can be supposed that NaCl is present as an impurity from the TEMPO treatment, however the exceeding Na could come from sodium carboxylate, also highlighted by FTIR.

**Figure 6:**
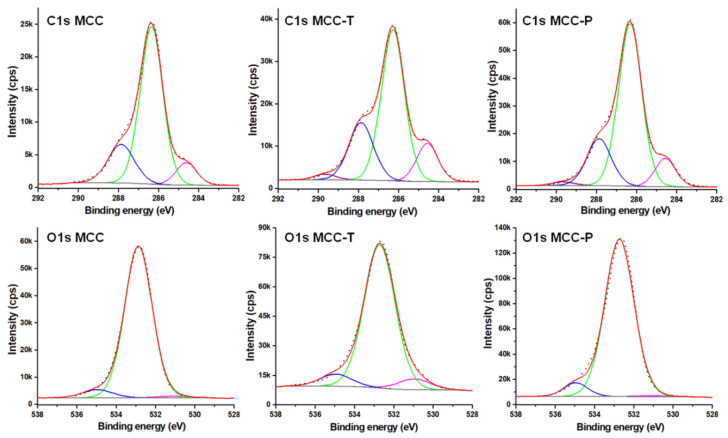
High resolution XPS spectra and deconvolution in the C1s and O1s regions.

High resolution XPS spectra for C1s and O1s regions are shown in Figure 6. The C1s peak was fitted with 3 components in the case of MCC and 4 components for treated cellulose (Table 2): C_1_ corresponding to C–C or C–H at a binding energy of 284.6 eV, C_2_ assigned to a carbon bonded to a single oxygen in alcohol or ether groups (C–O) at a binding energy of 286.3 eV, C_3_ ascribed to O–C–O or C=O at 288.0 eV, and C_4_ in O–C=O at 289.7 eV [39,40]. The C3/C2 ratio, 0.29, 0.41 and 0.31 for MCC, MCC-T and MCC-P, is higher than the theoretical value (0.2), showing the presence of carbonyl groups and a higher degree of oxidation, especially for MCC-T. In contrast to MCC, both treated celluloses contain C_4_ species (i.e., carboxyl or carboxylate groups) but MCC-T in a higher proportion. In general, the TEMPO treatment of cellulose led to a much higher degree of oxidation than the plasma one, which is obvious from the higher increase of C_3_ and C_4_ proportion at the expense of C_2_ in this case. Similar trends were reported by Coseri et al. for differently oxidized microcrystalline cellulose [38].

Some more information can be obtained from the deconvolution of O1s in high resolution XPS spectra (Figure 6). To O1s region contributed: O_1_, double bonded O (C=O) in sodium carboxylate and other oxidized groups (aldehydes, ketones) at 531.0 eV, O_2_, single bonded O from C-OH and C–O–C of pyranose ring at 532.9 eV and O_3_, which can be assigned to carboxylic acid functional groups and water, at 535.0 eV [40,41,42]. The relative atomic concentrations of these components (Table 2) undoubtedly show that the plasma treatment induced a small increase of carboxylic groups on cellulose, ensuring a slight oxidation while the TEMPO treatment determined a significant oxidation of cellulose, especially as carboxylate and carboxylic groups. The cellulose surface changes observed by XPS are in good agreement and strengthen the results obtained by FTIR.

### 3.2. Thermal Properties of PHB Composites with Different Treated MCC

**Figure 7:**
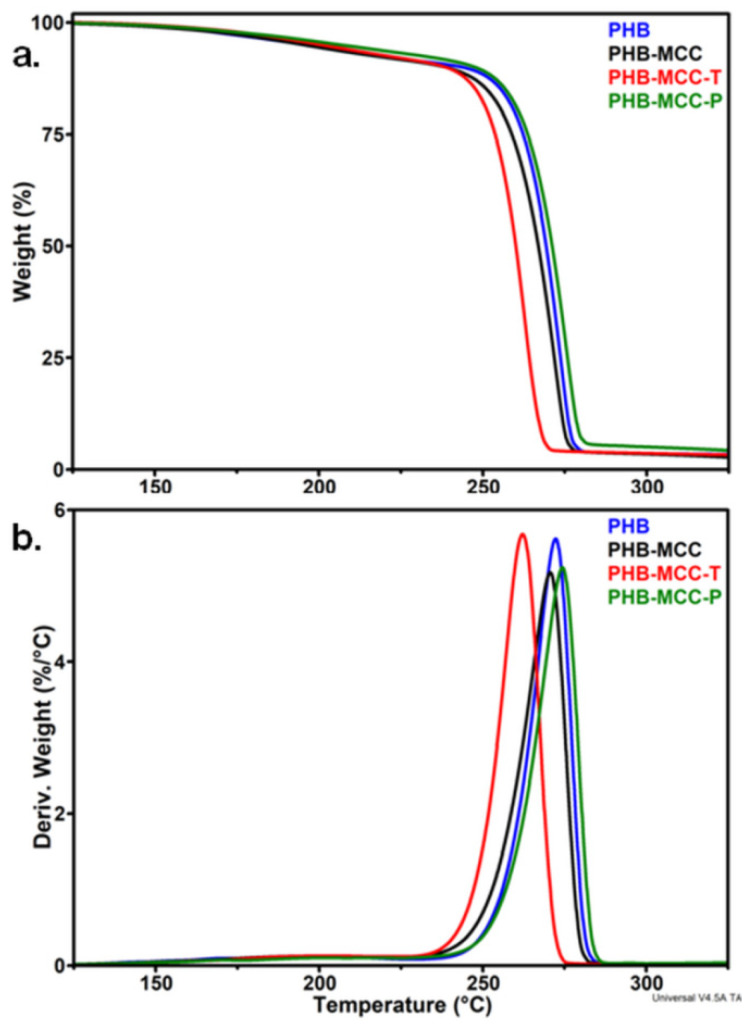
TGA (**a**) and DTG (**b**) curves of PHB composites with untreated and treated MCC.

Untreated and surface treated celluloses were used as reinforcement in PHB. Figure 7 shows the TGA and derivative (DTG) curves of PHB composites containing 2 wt % MCC, MCC-T, and MCC-P. Although the effect of different treated MCC on the thermal stability of PHB is similar to the effect of the treatments on cellulose (Figure 3), meaning an increased stability for MCC-P and a decreased one for MCC-T, the difference between the characteristic temperatures of the composites is much attenuated (Table 3).

The addition of MCC in PHB decreases the *T_on_* and *T_d_*, with only 2–3 °C, which is a common effect of cellulose fillers in PHB [7]. One cause of the increased degradation was supposed to be the higher thermal conductivity of cellulose compared to PHB [12]. Impurities, such as increased melt viscosity after the incorporation of cellulose in the polymer matrix or the presence of bond water in cellulose, were also considered as sources of an enhanced thermal degradation of PHB after the addition of cellulose [7].

The addition of MCC-T decreased the *T_on_* and *T_d_* of PHB composites with 9–10 °C. The presence of oxidized groups on the surface of MCC-T may enhance not only the thermal degradation of cellulose but also the degradation of PHB because they may catalyze the depolymerization reaction of PHB by interacting with the carboxyl group and favoring the chain scission reaction [43]. Considering that the melt processing took place at about 170 °C, the effect of oxidized compounds might be much enhanced at this temperature. Moreover, XPS survey spectra (Figure 5) and FTIR showed the presence of Na as sodium carboxylate on the surface of MCC-T which may catalyze the depolymerization reaction of PHB. Indeed, previous study has shown that many metal compounds but especially Na and Mg accelerate the random chain scission and the degradation of PHB [44].

In contrast to MCC and MCC-T, the addition of MCC-P slightly increased the *T_on_* and *T_d_* of PHB composites with 1–2 °C. Plasma treated MCC led to a better thermal behavior of PHB-MCC-P composite because of the mild oxidation of cellulose following the plasma treatment in contrast to the harsh TEMPO oxidation, also illustrated by the FTIR results. Moreover, the cleaning of cellulose during the submerged plasma treatment removes metals or other impurities which may catalyze the decomposition of PHB at high temperature [44]. It is remarkable that the thermal stability of composites at processing temperature, illustrated by the temperature at 5% weight loss (*T*_5%_), was much improved in PHB composites containing treated MCC (Table 3). An increase of *T*_5%_ with about 11 °C in the case of PHB-MCC-P and 5 °C in the case of PHB-MCC-T was noticed.

**Figure 8:**
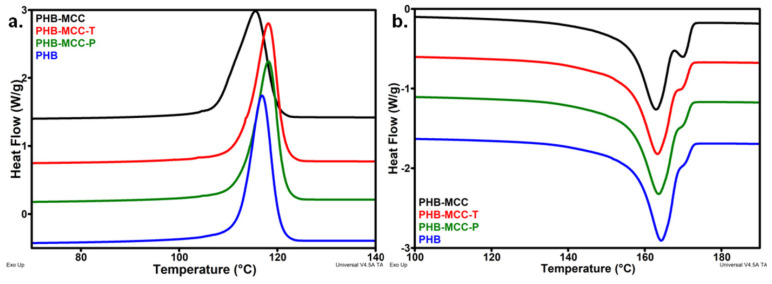
DSC thermograms of PHB and composites for the first (**a**) and second heating (**b**).

The DSC thermograms during the cooling and second heating cycles are shown in Figure 8 and the corresponding thermal parameters are listed in Table 4. Important differences between the crystallization behaviors of composites with different treated MCC were noticed in the cooling cycle (Figure 8a).

As observed in Figure 8a, The *T_c_* of PHB slightly increased in the composites with treated MCC and decreased in the composite with untreated cellulose. Although the difference between the composites with untreated and treated cellulose is at most 3 °C, this reflects the influence of MCC treatment on the crystallization of PHB. Moreover, the width at half-height of crystallization peak was almost double in the case of PHB-MCC compared to PHB and the composites with treated MCC. The broader crystallization peak and the lower *T_c_* show that MCC hindered the crystallization of PHB and slowed down the crystallization rate. Previous study has shown that nanocellulose is a good nucleating agent in PHB, increasing the crystallization rate [7]. Therefore, the effect of MCC on the crystallization of PHB may be due to the poor interface with the polymer and the micrometric size of cellulose fibers. On the contrary, the good influence of treated fibers suggests a better interface between PHB and MCC-T or MCC-P. In addition, both plasma and TEMPO treatments contributed to the defibrillation of cellulose, reducing the size of the fibers, as pointed out by previous studies [16,21].

Similar behavior was observed in the second heating cycle, with a main melting peak and a shoulder at a higher temperature (Figure 8b). The melting temperature remained unchanged after the addition of cellulose, regardless the treatment. However, the crystallinity followed the same trend as observed in the cooling cycle, a slight decrease in the composite with untreated MCC and a slight increase in that containing treated celluloses. This may be an effect of a better dispersion of treated MCC in PHB and better interactions between PHB and plasma or TEMPO treated cellulose. SEM analysis may enlighten this aspect.

### 3.3. SEM Images of PHB Composites with Treated MCC

**Figure 9:**
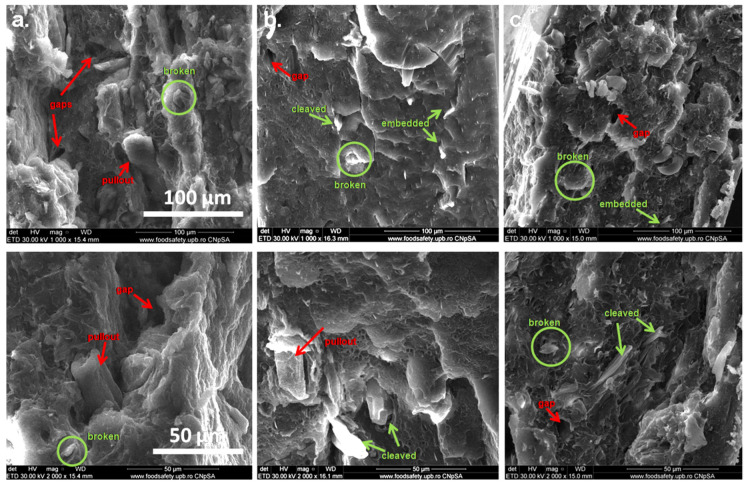
SEM images of PHB composites with different treated MCC: untreated MCC ((**a**); ×1000, ×2000); MCC-T ((**b**); ×1000, ×2000); MCC-P ((**c**); ×1000, ×2000).

The SEM images in Figure 9 show the surface morphology of the fractured PHB composites with different treated cellulose.

Both well embedded broken fibers and pullout fibers and gaps were observed in all the samples but the frequency of debonded fibers and gaps was higher in PHB-MCC than in the composites with treated fibers. Notably, the presence of fibers broken on the transverse section indicates a good PHB–cellulose interface, and such fibers were frequently observed in the composites with TEMPO and plasma modified fibers. This shows good bonding between the polymer and cellulose fibers, which may be benefic to their mechanical properties. Similarly, thinner submicron fibers were mostly observed in the composites with MCC-T and MCC-P, because both TEMPO oxidation and plasma treatment decrease the size of original cellulose fibers and caused defibrillation [13,21]. A better dispersion of fibers was noticed in the case of PHB-MCC-P.

### 3.4. DMA Analysis of PHB Composites with Treated MCC

**Figure 10:**
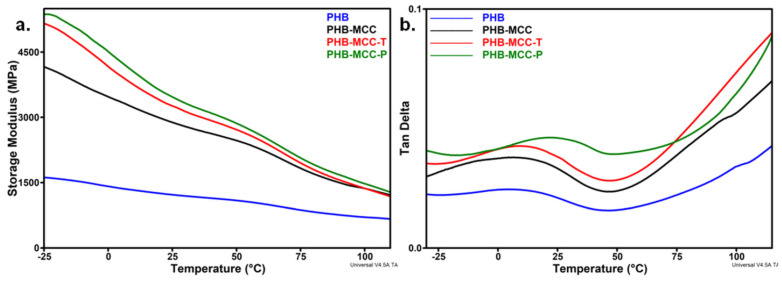
Storage modulus (**a**) and tan δ (**b**) vs temperature curves for PHB and composites with different treated MCC.

The stiffness of PHB composites as function of temperature was investigated by DMA (Figure 10). The addition of untreated and treated MCC in PHB led to an increase of the storage modulus (*E’*) (Figure 10a). However, the increase was more accentuate (2–3 times) for the composites with treated MCC, especially with plasma treated fibers. An increase of the *E’* by 184% for PHB-MCC-P and 167% for PHB-MCC-T was noticed at room temperature (Table 5).

The more accentuated decrease of the *E’*, observed between about −10 °C and 25–30 °C, was determined by the transition of PHB from glassy to rubbery state and the activation of molecular segmental motions at the glass transition temperature [44]. Variation of loss factor (tan δ) with temperature (Figure 10b) emphasizes the relative contributions of the elastic and viscous components when temperature increases. A shift of the glass transition temperature (*T_g_*) to a higher temperature was observed for all the composites compared to PHB, however the highest shift, of 18 °C, was observed for PHB-MCC-P. This shows a good interface and a uniform dispersion of MCC-P in the polymer matrix. Plasma treatment induced a mild oxidation on the surface of cellulose, as indicated by both FTIR and XPS results, and the formation of carboxylate groups may increase the compatibility between poly(hydroxyl butyric acid) matrix and COOH bearing cellulose. The influence of MCC-T was not so important, probably because of the opposite effects of surface functionalization and degradation. TEMPO oxidation induces the surface functionalization of cellulose, improving the interface and mechanical properties, but the presence of metal compounds and other impurities from the chemical treatment enhances the decomposition of PHB, thus decreasing the molecular weight and the mechanical properties [7,45]. This is supported by the lower thermal stability of this composite (Figure 7). As a result, the increase of the mechanical properties in PHB-MCC-T was not as high as in the case of PHB-MCC-P, although TEMPO oxidation induced a much strong surface modification of cellulose.

## 4. Conclusions

Microcrystalline cellulose was surface modified by a plasma treatment in liquid using a Y-shaped tube and TEMPO mediated oxidation. The plasma treatment of water suspensions of cellulose ensured an increased flow of the oxidative gas, being a simple and highly reproducible method for cellulose modification. FTIR and XPS results showed that both treatments led to the surface functionalization of cellulose. However, TEMPO oxidation had a much stronger effect, leading to a decrease of thermal stability of MCC by 80 °C. Plasma and TEMPO modified celluloses were incorporated in a PHB matrix and they influenced the morphology and thermal and mechanical properties of composites. Both treatments improved the fiber-polymer interface and the mechanical properties of PHB composites. An increase of the storage modulus by184% in PHB-MCC-P and 167% in PHB-MCC-T was noticed at room temperature. The increase of the mechanical properties in the composite with TEMPO oxidized cellulose was not as high as in the case of that containing plasma treated cellulose, although the chemical treatment induced a much stronger surface modification of cellulose. The main cause of this behavior is, probably, the significant degradation of MCC following TEMPO oxidation. The PHB composites containing plasma modified cellulose are promising materials for biomedical applications. In addition, the filamentary Y-shaped DBD plasma jet immersed in the liquid suspension of cellulose is an environmentally friendly, simple, and cheap treatment, which is proposed as an alternative to the mechano-chemical methods usually employed for the modification of cellulose.

## Figures and Tables

**Table 1:** The elemental surface composition, in atomic %, from XPS survey spectra before (MCC) and after the treatments (MCC-T and MCC-P).

Samples	C1s (%)	O1s (%)	Na1s (%)	Cl 2p (%)	O/C
MCC	57.3	42.7	-	-	0.75
MCC-T	52.8	39.4	5.3	2.5	0.75
MCC-P	56.9	43.1	-	-	0.76

**Table 2:** The relative atomic concentrations of differently bound carbon and oxygen atoms as determined from high resolution XPS spectra.

Samples	C1s				O1s		
	C_1_ (%)	C_2_ (%)	C_3_ (%)	C_4_ (%)	O_1_ (%)	O_2_ (%)	O_3_ (%)
MCC	9.4	70.3	20.3	-	1.1	93.4	5.5
MCC-T	14.0	59.6	24.3	2.1	6.7	85.7	7.6
MCC-P	10.5	67.4	20.6	1.4	0.6	92.7	6.8

**Table 3:** TGA results for PHB composites with different treated MCC.

Characteristic Temperatures	PHB	PHB-MCC	PHB-MCC-T	PHB-MCC-P
*T*_5%_, °C	195.6	195.7	200.9	206.8
*T*_on_, °C	262.6	259.4	253.7	263.6
*T*_d_, °C	272.3	270.6	262.1	274.3

**Table 4:** DSC data collected from the heating-cooling-heating DSC curves of composites.

Composites	PHB	PHB-MCC	PHB-MCC-T	PHB-MCC-P
*T*_c_, °C	116.9	115.5	118.2	118.2
∆*H*_c_	76.6	74.0	77.1	76.9
*T*_m_, °C	164.2	163.0	163.3	163.6
∆*H*_m_	81.1	78.4	81.5	81.5
*X*_c_, %	55.5	54.8	57.0	57.0

∆*H*_c_, *T*_c_—crystallization enthalpy and crystallization temperature. ∆*H*_m_, *T*_m_—melting enthalpy and melting temperature.

**Table 5:** DMA data for PHB and composites: PHB-MCC, PHB-MCC-T and PHB-MCC-P.

Composites	PHB	PHB-MCC	PHB-MCC-T	PHB-MCC-P
*T_g_*, °C	3.8	5.8	9.2	21.8
*E’* (−25 °C), MPa	1617	4164	5160	5370
*E’* (25 °C), MPa	1221	2885	3264	3470
*E’* (75 °C), MPa	871	1830	1952	2068
